# Maternal dietary fiber intake during pregnancy and child development: the Japan Environment and Children's Study

**DOI:** 10.3389/fnut.2023.1203669

**Published:** 2023-07-27

**Authors:** Kunio Miyake, Sayaka Horiuchi, Ryoji Shinohara, Megumi Kushima, Sanae Otawa, Hideki Yui, Yuka Akiyama, Tadao Ooka, Reiji Kojima, Hiroshi Yokomichi, Kazuki Mochizuki, Zentaro Yamagata, The Japan Environment Children's Study Group, Michihiro Kamijima

**Affiliations:** ^1^Department of Health Sciences, Interdisciplinary Graduate School of Medicine and Engineering, University of Yamanashi, Chuo, Japan; ^2^Center for Birth Cohort Studies, University of Yamanashi, Chuo, Japan; ^3^Department of Local Produce and Food Sciences, Faculty of Life and Environmental Sciences, University of Yamanashi, Kofu, Japan

**Keywords:** DOHaD (Developmental Origins of Health and Disease), dietary fiber, birth cohort, FFQ food frequency questionnaire, ASQ-3, gut microbiome

## Abstract

**Background:**

Animal studies have shown that maternal low-fiber diets during pregnancy may impair brain development and function in offspring, but this has not been validated by epidemiological studies. The aim of this study was to investigate the link between maternal dietary fiber intake during pregnancy and neurodevelopmental delay in offspring using a large birth cohort.

**Methods:**

A total of 76,207 mother-infant pairs were analyzed using data from the Japan Environment and Children's Study, a nationwide prospective cohort study. Maternal dietary fiber intake was estimated using the food frequency questionnaire in mid-pregnancy. Maternal dietary fiber intake was adjusted for energy and classified into quintiles. Developmental delay was assessed in five domains using the Japanese version of the Ages and Stages Questionnaire, Third Edition at the age of 3 years. The logistic regression analysis was performed to estimate the odds ratio (OR) and 95% confidence interval (CI) for the link between dietary fiber intake during pregnancy and developmental delay at the age of 3 years.

**Results:**

The lowest intake group of total dietary fiber had a higher risk of delayed communication [adjusted OR (aOR), 1.51; 95% CI, 1.32–1.74], fine motor (aOR, 1.45; 95% CI, 1.32–1.61), problem-solving (aOR, 1.46; 95% CI, 1.32–1.61), and personal-social skills (aOR, 1.30; 95% CI, 1.12–1.50) than did the highest intake group. An analysis that excluded the effects of insufficient folic acid intake during pregnancy also showed a similar trend.

**Conclusion:**

This study showed that maternal dietary fiber deficiency during pregnancy might influence an increased risk of neurodevelopmental delay in offspring.

## 1. Introduction

The Developmental Origins of Health and Disease concept (DOHaD) has proposed that various environmental factors (e.g., undernutrition, stress, and chemical exposures) during the fetal period and infancy are linked with increased risk of several diseases (e.g., obesity, diabetes, allergic diseases, and neurodevelopmental diseases) later in life (1, 2). The first 1,000 days of life, from conception to a child's second birthday, are considered particularly important for child development. Intervention programs are being implemented in developing countries to improve malnutrition (3). Maternal malnutrition during pregnancy is a serious problem even in developed countries. In Japan, according to the Dietary Reference Intakes for Japanese (2020 version), the intake of several nutrients (e.g., dietary fiber, vitamin C, folic acid, and iron) is well-below the requirements for pregnant women (4). In young women, underweight, diet orientation, low-protein diet, and high-fat diet are also problems (5).

Dietary fiber is defined as edible carbohydrate polymers that are not decomposed by human digestive enzymes, and are classified into soluble and insoluble fiber depending on the difference in water solubility. Epidemiological studies have shown that increasing dietary fiber consumption is linked to a lower risk of various chronic diseases, including type 2 diabetes (6), cancer (7), and cardiovascular disease (8). Animal studies have reported that a high-fiber diet during pregnancy is linked to improved immune system development, decreased metabolic syndrome, and decreased cognitive and social dysfunction caused by maternal obesity in offspring (9–11). Another animal study also showed that a low-fiber diet during pregnancy impairs brain nerve function in offspring (12). However, no epidemiological studies have confirmed a link between maternal dietary fiber intake during pregnancy and neurodevelopment in offspring. Therefore, we investigated whether maternal dietary fiber intake during pregnancy was linked to child developmental delay at the age of 3 years.

## 2. Materials and methods

### 2.1. Study setting and population

**Figure 1:**
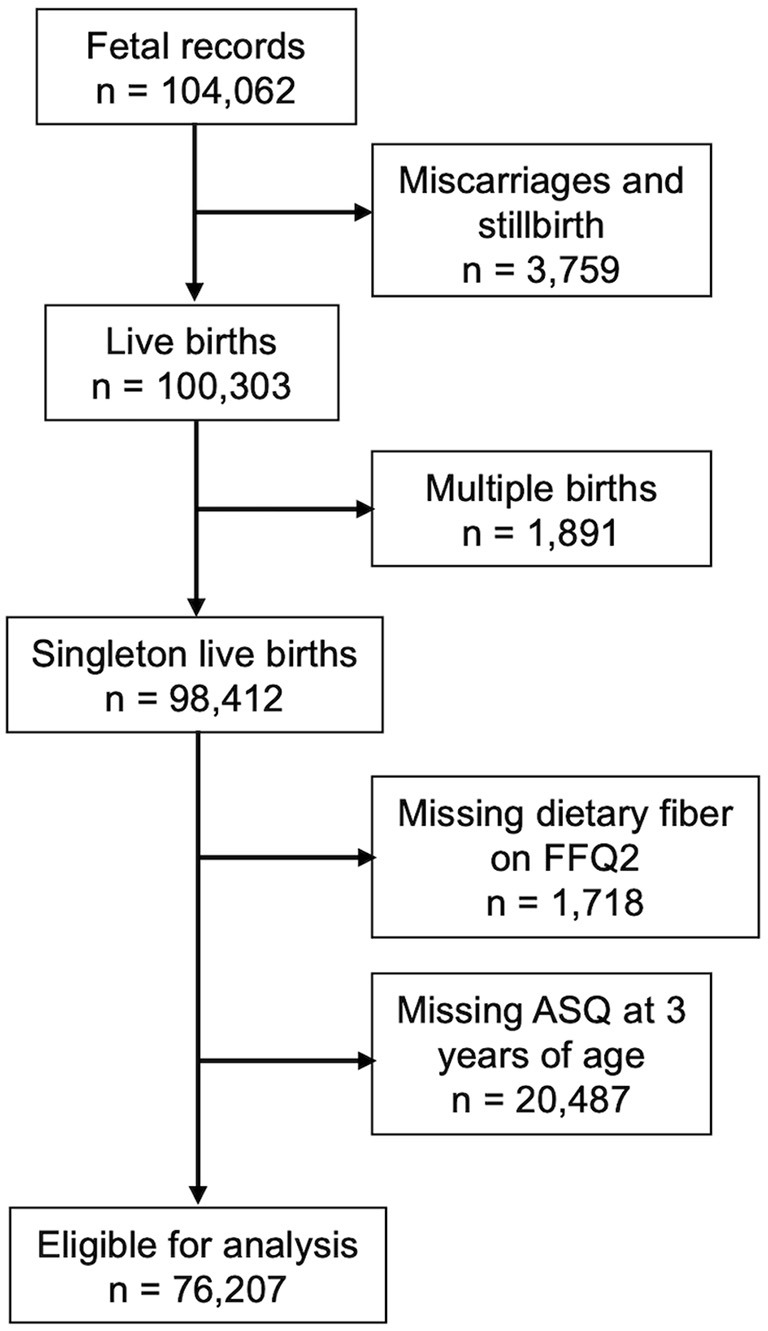
Flow chart of the participants.

The Japan Environment and Children's Study (JECS) is an ongoing nationwide prospective birth cohort study. The detailed protocols have been published elsewhere (13, 14). Participants were recruited between January 2011 and March 2014 in 15 Regional Centers covering 19 prefectures across Japan. We used the dataset jecs-ta-20190930-qsn, which was first released in October 2019 and completed in March 2022. We eliminated 3,759 miscarriages or stillbirths and 1,891 multiple pregnancies from a total of 104,062 fetal records. In addition, 1,718 and 20,487 were not included in this study because Food Frequency Questionnaire (FFQ) in mid-pregnancy and the Japanese translations of Ages and Stages Questionnaires, Third Edition (J-ASQ-3) at 3 years, respectively, had missing data. Finally, 76,207 mother-child pairs were analyzed in this study (Figure 1).

The JECS protocol was reviewed and approved by the Ministry of the Environment's Institutional Review Board on Epidemiological Studies and the Ethics Committees of all participating institutions (ethical approval number: 100910001). The JECS was conducted following the Declaration of Helsinki, and written informed consent was obtained from all the participants.

### 2.2. Dietary information

Dietary information was obtained using the FFQ, which is a validated self-administered dietary questionnaire evaluated in previous studies (15). Respondents were asked about their dietary status from conception to the answering date Respondents were asked about their dietary status during the second/third trimester. The food and nutrient intakes were calculated using the FFQ items based on the 2010 edition of the Standardized Tables of Food Composition in Japan. Energy adjustment for nutrients was conducted using the nutrient density model (per 1,000 kcal) (16). Participants were categorized into quintiles based on dietary fiber intake. The lowest and highest quintiles were Q1 and Q5, respectively.

To analyze the effects of folic acid intake, we used data on dietary folic acid intake and the frequency of folic acid supplement use during the second and third trimesters. Based on the estimated mean folic acid requirement for pregnant Japanese women (400 μg/day) (4), we divided the population into two groups. Mothers answered about folate supplement intake during pregnancy by choosing frequency from never, once a month, 2–3 times a month, 1–3 times a week, 4–6 times a week, once a day, and twice or more times a day. Furthermore, we recategorized it into three groups: none, occasional (less than six times per week), and daily (more than once a day).

### 2.3. Outcome definitions

Developmental delays at the age of 3 years were assessed using the ASQ-3, which was included in the questionnaire sent when the child was 3 years old. Parents or caregivers filled out the ASQ-3 developmental screening questionnaire (17). The ASQ-3 comprised 30 questions across the following five domains: communication, gross motor, fine motor, problem-solving, and personal-social skills. A respondent chose yes, sometimes, or no for each question and scored 10 for yes, 5 for sometimes, and 0 for no. The J-ASQ-3, which was used in this study, has been validated using benchmark scores for Japanese children (17). The child was determined to have a developmental delay in that domain if their score fell below the cutoff in each domain.

### 2.4. Covariates

Covariates were selected a priori based on previous studies (18–20). Information on maternal smoking and drinking during pregnancy, annual household income (million yen), maternal educational level, maternal age at birth, and older siblings was collected from mothers in the first and second/third trimesters of pregnancy using a self-administered questionnaire. Child sex, pre-pregnancy body mass index (BMI), mode of delivery, birth weight, and the gestational week at birth were extracted from medical record transcripts. Preterm birth was defined as delivery at < 37 weeks gestation. Maternal age at birth was classified as < 25, 25–29, 30–34, and ≥35 years. Pre-pregnancy BMI was categorized into three groups: underweight (< 18.5 kg/m^2^), normal (18.5–24.9 kg/m^2^), and overweight (≥25 kg/m^2^). Information on childcare facility attendance, caretaker other than the child's mother, and breastfeeding was collected from the self-questionnaire administered to mothers when their children were 1 year old. In this questionnaire, the mother was asked, “I love this child”, to which she chose “Strongly agree”; “Somewhat agree”; “Somewhat disagree”; or “Strongly disagree”. We re-categorized the responses into two categories: agree and disagree.

The Kessler Psychological Distress Scale (K6) score was extracted from a self-questionnaire during the second trimester and when the child reached 1 year of age. Scores above the cutoff points of K6 (≥5 points) have been reported to indicate poor mental health in Japanese (21).

### 2.5. Statistical analyses

The logistic regression analysis was used to estimate the crude and adjusted odds ratios (cOR and aOR, respectively), as well as the 95% confidence interval (CI), for the link between dietary fiber intake during pregnancy and developmental delay in children at the age of 3 years. Multivariable models were adjusted for pre-pregnancy BMI, maternal education level, maternal smoking and drinking during pregnancy, annual household income, older siblings, maternal age at birth, gestational age at birth, birth weight, child's sex, attendance at a childcare facility at the age of 1 year, and breastfeeding until the age of 1 year, maternal mental health during pregnancy and when her child was 1 year of age, caretaker other than the child's mother at 1 year of age, and attachment bond when her child was 1 year of age. Missing values were excluded from the multivariate analysis. A population with sufficient folic acid intake was used for sensitivity analysis. Furthermore, the median value of each intake quintile was used to calculate P for the trend. A P = 0.05 (two-sided) was considered significant. All statistical analyses were performed using the Statistical Package for the Social Sciences software version 27.

## 3. Results

The characteristics of the mothers and their children are presented in Table 1. Mean [± standard deviation (SD)] intakes of total, soluble, and insoluble dietary fiber (g/1,000 kcal/day) during mid-pregnancy were 6.18 ± 2.04, 1.50 ± 0.58, and 4.45 ± 1.40, respectively. The numbers of children aged 3 years, whose scores fell below the threshold for each J-ASQ-3 category were as follows: 3,125 (4.1%) for communication, 3,527 (4.6%) for gross motor, 5,866 (7.7%) for fine motor, 5,696 (7.5%) for problem-solving, and 2,594 (3.4%) for personal-social skills.

**Table 1:** Characteristics of the study population.

**Variable**	**Mean ±SD or *N* (%)**
**Intake of dietary fiber during mid-pregnancy (g/1,000 kcal/day)**	
Total dietary fiber	6.18 ± 2.04
Soluble dietary fiber	1.50 ± 0.58
Insoluble dietary fiber	4.45 ± 1.40
**Folic acid diet during mid-pregnancy (**μ**g/day)**	259.48 ± 158.09
< 400	67,257 (88.3)
≥400	8,950 (11.7)
**Folic acid supplement during pregnancy**	
None	39,116 (51.3)
Occasional	15,318 (20.1)
Daily	15,976 (21.0)
Missing data	5,797 (7.6)
**Maternal age at birth (years)**	
< 25	6,277 (8.2)
25–29	20,681 (27.1)
30–34	27,674 (36.3)
≥35	21,574 (28.3)
Missing data	1 (0.0)
**Maternal smoking status during pregnancy**	
No	67,717 (88.9)
Yes	2,457 (3.2)
Missing data	6,033 (7.9)
**Maternal drinking during pregnancy**	
No	67,561 (88.7)
Yes	1,834 (2.4)
Missing data	6,812 (8.9)
**Pre-pregnancy BMI (kg/m** ^2^ **)**	
< 18.5	12,392 (16.3)
18.5–25	56,307 (73.9)
>25	7,470 (9.8)
Missing data	38 (0.0)
**Maternal education level (years)**	
≤ 12	23,374 (30.7)
>12	46,942 (61.6)
Missing data	5,930 (7.8)
**Annual household income (millions of yen)**	
< 4	24,895 (32.7)
≥4	41,240 (54.1)
Missing data	10,072 (13.2)
**Birth weight (g)**	
< 2,500	5,966 (7.8)
Missing data	208 (0.3)
**Gestational age at birth (week)**	
≥37	72,667 (95.4)
< 37	3,378 (4.4)
Missing data	162 (0.2)
**Mode of delivery**	
Vaginal	61,877 (81.2)
Cesarean section	14,011 (18.4)
Missing data	319 (0.4)
**Child sex**	
Male	39,053 (51.2)
Female	37,154 (48.8)
**Older siblings**	
No	35,211 (46.2)
Yes	40,736 (53.5)
Missing data	260 (0.3)
**Mother's K6 scores during pregnancy**	
0–4 points	55,215 (72.5)
≥5 points	20,832 (27.3)
Missing data	160 (0.2)
**Mother's K6 scores when her child was 1 year of age**	
0–4 points	58,645 (77.0)
≥5 points	16,091 (21.1)
Missing data	1,471 (1.9)
**Attendance to nursery school at 1 year of age**	
No	55,268 (72.5)
Yes	19,296 (25.3)
Missing data	1,643 (2.2)
**Breastfeeding at 1 year of age**	
No	28,799 (37.8)
Yes	45,625 (59.9)
Missing data	1,783(2.3)
**Caretaker other than the child's mother when her child was 1**	
**year of age**	
No	2,953 (3.9)
Yes	68,400 (89.8)
Missing data	4,854 (6.4)
**Attachment bond when her child was 1 year of age**	
Strongly agree	71,271 (93.5)
Somewhat agree	3,324 (4.4)
Somewhat disagree	87 (0.1)
Strongly disagree	30 (0.0)
Missing data	1,495(2.0)
**ASQ score at age 3 years**	
**Communication skill**	
≥29.95	73,082 (95.9)
< 29.95	3,125 (4.1)
**Gross motor skill**	
≥39.26	72,680 (95.4)
< 39.26	3,527 (4.6)
**Fine motor skill**	
≥27.91	70,341 (92.3)
< 27.91	5,866 (7.7)
**Problem-solving skill**	
≥30.03	70,511 (92.5)
< 30.03	5,696 (7.5)
**Personal-social skill**	
≥29.89	73,613 (96.6)
< 29.89	2,594 (3.4)

ASQ, Ages and Stages Questionnaires; BMI, body mass index; SD, standard deviation.

Table 2 shows the association between maternal total dietary fiber intake during mid-pregnancy and developmental delay in children at the age of 3 years. In comparison to the highest intake group, the lower intake groups of total dietary fiber had a higher risk of children showing developmental delays in four domains. For instance, the lowest intake group of total dietary fiber had the following aOR and 95% CI: communication (aOR, 1.51; 95% CI, 1.32–1.74; *P* for trend < 0.001), fine motor (aOR, 1.45; 95% CI, 1.32–1.61; *P* for trend < 0.001), problem-solving (aOR, 1.46; 95% CI, 1.32–1.61; *P* for trend < 0.001), and personal-social skills (aOR, 1.30; 95% CI, 1.12–1.50; *P* for trend < 0.001).

**Table 2:** Association between intake of total dietary fiber on mid-pregnancy and development delay at the age of 3 years.

**Subscales in ASQ-3**	**Communication**		**Gross motor**		**Fine motor**		**Problem solving**		**Personal-social**	
	**cOR (95% CI)**	**aOR (95% CI)**	**cOR (95% CI)**	**aOR (95% CI)**	**cOR (95% CI)**	**aOR (95% CI)**	**cOR (95% CI)**	**aOR (95% CI)**	**cOR (95% CI)**	**aOR (95% CI)**
**Total dietary fiber (g/1,000 kcal/day)**										
Q5 (≥7.56)	1.00	1.00	1.00	1.00	1.00	1.00	1.00	1.00	1.00	1.00
Q4 (6.39– < 7.56)	**1.14 (1.01–1.29)**	**1.19 (1.04–1.37)**	0.99 (0.89–1.10)	1.03 (0.91–1.17)	1.09 (1.00–1.19)	**1.11 (1.01–1.23)**	1.04 (0.95–1.14)	1.07 (0.97–1.19)	1.11 (0.98–1.27)	1.13 (0.97–1.30)
Q3 (5.51– < 6.39)	**1.26 (1.12–1.42)**	**1.31 (1.15–1.51)**	1.05 (0.94–1.16)	1.10 (0.97–1.24)	**1.17 (1.07–1.28)**	**1.17 (1.06–1.29)**	**1.21 (1.11–1.32)**	**1.23 (1.12–1.36)**	1.12 (0.98–1.27)	1.11 (0.96–1.29)
Q2 (4.62– < 5.51)	**1.37 (1.22–1.54)**	**1.41 (1.23–1.61)**	1.03 (0.93–1.15)	1.07 (0.95–1.21)	**1.25 (1.15–1.37)**	**1.24 (1.12–1.37)**	**1.26 (1.16–1.38)**	**1.30 (1.17–1.44)**	**1.21 (1.07–1.37)**	**1.26 (1.09–1.46)**
Q1 (< 4.62)	**1.48 (1.32–1.66)**	**1.51 (1.32–1.74)**	1.07 (0.96–1.19)	1.13 (1.00–1.28)	**1.50 (1.38–1.64)**	**1.45 (1.32–1.61)**	**1.43 (1.31–1.55)**	**1.46 (1.32–1.61)**	**1.33 (1.17–1.51)**	**1.30 (1.12–1.50)**
*P* for trend	**< 0.001**	**< 0.001**	0.186	**0.043**	**< 0.001**	**< 0.001**	**< 0.001**	**< 0.001**	**< 0.001**	**< 0.001**

cOR, crude odds ratio; aOR, adjusted odds ratio; CI, confidence interval; ASQ-3, Ages and Stages Questionnaires, Third Edition. Boldface indicates significance (*p* < 0.05).

Adjusted for maternal smoking and drinking during pregnancy, maternal age at birth, pre-pregnancy BMI, maternal and paternal education levels, annual household income, child sex, mode of delivery, birthweight, gestational age at birth, older siblings, attendance a childcare facility at 1 year of age, breastfeeding until 1 year of age, mother's K6 scores during pregnancy and when her child was 1 year of age, caretaker other than the child's mother at 1 year of age, and attachment bond when her child was 1 year of age.

Table 3 shows the association between maternal intake of soluble and insoluble dietary fiber during mid-pregnancy and child developmental delay at age 3. Similar to the results for total dietary fiber, both low-fiber groups were linked to a higher risk of child developmental delay in four domains, excluding gross motor skill. For gross motor skills, the lower soluble dietary fiber groups had a higher risk of child developmental delay than did the groups with highest soluble dietary fiber. (Q1: aOR, 1.15; 95% CI, 1.02–1.31; Q2: aOR, 1.18; 95% CI, 1.04–1.33; Q3: aOR, 1.16; 95% CI, 1.02–1.31; Q4: aOR, 1.16; 95% CI, 1.02–1.31; *P* for trend = 0.016).

**Table 3:** Association between intake of soluble and insoluble dietary fiber on mid-pregnancy and development delay at the age of 3 years.

**Subscales in ASQ-3**	**Communication**	**Gross motor**	**Fine motor**	**Problem solving**	**Personal-social**
	**aOR (95% CI)**	**aOR (95% CI)**	**aOR (95% CI)**	**aOR (95% CI)**	**aOR (95% CI)**
**Soluble dietary fiber (g/1,000 kcal/day)**					
Q5 (≥1.89)	1.00	1.00	1.00	1.00	1.00
Q4 (1.55– < 1.89)	**1.17 (1.02–1.34)**	**1.16 (1.02–1.31)**	1.07 (0.96–1.18)	1.05 (0.95–1.17)	1.11 (0.96–1.28)
Q3 (1.31– < 1.55)	**1.24 (1.08–1.42)**	**1.16 (1.02–1.31)**	**1.11 (1.00–1.23)**	**1.21 (1.09–1.34)**	1.15 (1.00–1.33)
Q2 (1.06– < 1.31)	**1.43 (1.26–1.64)**	**1.18 (1.04–1.33)**	**1.23 (1.11–1.36)**	**1.32 (1.19–1.46)**	**1.26 (1.09–1.45)**
Q1 (< 1.06)	**1.49 (1.30–1.70)**	**1.15 (1.02–1.31)**	**1.36 (1.23–1.50)**	**1.40 (1.27–1.55)**	**1.22 (1.05–1.41)**
*P* for trend	**< 0.001**	**0.016**	**< 0.001**	**< 0.001**	**0.002**
**Insoluble dietary fiber (g/1,000 kcal/day)**					
Q5 (≥5.41)	1.00	1.00	1.00	1.00	1.00
Q4 (4.60– < 5.41)	**1.17 (1.02–1.35)**	0.99 (0.88–1.12)	1.10 (0.99–1.21)	1.03 (0.93–1.14)	1.07 (0.92–1.24)
Q3 (4.00– < 4.60)	**1.33 (1.16–1.53)**	**1.15 (1.02–1.29)**	**1.16 (1.05–1.29)**	**1.22 (1.11–1.35)**	1.12 (0.96–1.29)
Q2 (3.38– < 4.00)	**1.41 (1.23–1.61)**	1.04 (0.92–1.17)	**1.24 (1.12–1.37)**	**1.30 (1.17–1.43)**	**1.22 (1.06–1.41)**
Q1 (< 3.38)	**1.51 (1.31–1.73)**	1.12 (0.99–1.27)	**1.45 (1.32–1.61)**	**1.45 (1.31–1.60)**	**1.29 (1.11–1.49)**
*P* for trend	**< 0.001**	0.055	**< 0.001**	**< 0.001**	**< 0.001**

aOR, adjusted odds ratio; CI, confidence interval; ASQ-3, Ages and Stages Questionnaires, Third Edition. Boldface indicates significance (*p* < 0.05).

Adjusted for maternal smoking and drinking during pregnancy, maternal age at birth, pre-pregnancy BMI, maternal and paternal education levels, annual household income, child sex, mode of delivery, birthweight, gestational age at birth, older siblings, attendance a childcare facility at 1 year of age, breastfeeding until 1 year of age, mother's K6 scores during pregnancy and when her child was 1 year of age, caretaker other than the child's mother at 1 year of age, and attachment bond when her child was 1 year of age.

We also analyzed the association between maternal total dietary fiber intake during mid-pregnancy and child developmental delay at the age of 3 years, stratified by pre-pregnancy BMI (Table 4). In the normal BMI group, similar to the results presented in Table 2, a lower total dietary fiber intake was associated with a significantly higher risk of developmental delay at the age of 3 years in four domains (communication, fine motor, problem-solving, and personal-social skills). In the underweight BMI group, the lowest intake groups of total dietary fiber had a significantly higher risk of developmental delay in communication (Q1: aOR, 1.12; 95% CI, 1.07–2.15) and problem-solving (Q1: aOR, 1.50; 95% CI, 1.14–1.97) compared that of the group with the highest intake. In the overweight BMI group, the lowest intake groups of total dietary fiber had a significantly higher risk of developmental delay in communication (Q1: aOR, 1.64; 95% CI, 1.12–2.38), fine motor (Q1: aOR, 1.51; 95% CI, 1.15–2.00), and problem-solving skills (Q1: aOR, 1.59; 95% CI, 1.20–2.11) compared with that of group with the highest intake.

**Table 4:** Association between total dietary fiber intake on mid-pregnancy and development delay at the age of 3 years, stratified by pre-pregnancy BMI.

**Subscales in ASQ-3**	**Communication**	**Gross motor**	**Fine motor**	**Problem solving**	**Personal-social**
	**aOR (95% CI)**	**aOR (95% CI)**	**aOR (95% CI)**	**aOR (95% CI)**	**aOR (95% CI)**
**Pre-pregnancy BMI**<**18.5 (kg/m**^2^**)**					
Q5 (≥7.56)	1.00	1.00	1.00	1.00	1.00
Q4 (6.39– < 7.56)	1.10 (0.77–1.57)	0.90 (0.67–1.22)	0.96 (0.75–1.24)	1.25 (0.95–1.65)	0.96 (0.66–1.40)
Q3 (5.51– < 6.39)	1.07 (0.75–1.53)	1.14 (0.86–1.51)	0.93 (0.72–1.20)	1.35 (1.03–1.76)	0.91 (0.63–1.32)
Q2 (4.62– < 5.51)	1.20 (0.85–1.71)	1.11 (0.83–1.48)	1.08 (0.84–1.38)	1.52 (1.16–1.98)	0.98 (0.67–1.42)
Q1 (< 4.62)	**1.52 (1.07–2.15)**	1.03 (0.76–1.40)	1.09 (0.84–1.40)	**1.50 (1.14–1.97)**	1.16 (0.80–1.68)
*P* for trend	**0.022**	0.467	0.419	**0.001**	0.564
**Pre-pregnancy BMI 18.5–25 (kg/m** ^2^ **)**					
Q5 (≥7.56)	1.00	1.00	1.00	1.00	1.00
Q4 (6.39– < 7.56)	**1.21 (1.03–1.42)**	1.05 (0.91–1.21)	**1.15 (1.02–1.30)**	1.04 (0.92–1.17)	1.19 (1.00–1.41)
Q3 (5.51– < 6.39)	**1.32 (1.13–1.55)**	1.06 (0.92–1.22)	**1.23 (1.09–1.39)**	**1.23 (1.09–1.38)**	**1.22 (1.03–1.45)**
Q2 (4.62– < 5.51)	**1.43 (1.22–1.68)**	1.04 (0.90–1.21)	**1.30 (1.15–1.47)**	**1.28 (1.14–1.44)**	**1.39 (1.17–1.65)**
Q1 (< 4.62)	**1.50 (1.28–1.77)**	1.11 (0.96–1.29)	**1.53 (1.36–1.72)**	**1.42 (1.26–1.60)**	**1.39 (1.16–1.65)**
*P* for trend	**< 0.001**	0.199	**< 0.001**	**< 0.001**	**< 0.001**
**Pre-pregnancy BMI** >**25 (kg/m**^2^**)**					
Q5 (≥7.56)	1.00	1.00	1.00	1.00	1.00
Q4 (6.39– < 7.56)	1.20 (0.80–1.80)	1.15 (0.77–1.71)	1.10 (0.81–1.49)	1.08 (0.80–1.48)	1.03 (0.69–1.54)
Q3 (5.51– < 6.39)	**1.66 (1.13–2.45)**	1.36 (0.92–2.00)	1.19 (0.88–1.60)	1.14 (0.84–1.55)	0.83 (0.54–1.26)
Q2 (4.62– < 5.51)	**1.57 (1.07–2.31)**	1.24 (0.84–1.82)	1.09 (0.81–1.47)	1.16 (0.86–1.56)	1.02 (0.69–1.52)
Q1 (< 4.62)	**1.64 (1.12–2.38)**	1.39 (0.96–2.02)	**1.51 (1.15–2.00)**	**1.59 (1.20–2.11)**	1.08 (0.73–1.58)
*P* for trend	**0.004**	0.080	**0.005**	**0.001**	0.745

aOR, adjusted odds ratio; CI, confidence interval; ASQ-3, Ages and Stages Questionnaires, Third Edition. Boldface indicates significance (*p* < 0.05).

Adjusted for maternal smoking and drinking during pregnancy, maternal age at birth, maternal and paternal education levels, annual household income, child sex, mode of delivery, birthweight, gestational age at birth, older siblings, attendance a childcare facility at 1 year of age, breastfeeding until 1 year of age, mother's K6 scores during pregnancy and when her child was 1 year of age, caretaker other than the child's mother at 1 year of age, and attachment bond when her child was 1 year of age.

To exclude the effects of folic acid intake during pregnancy, we selected mothers who consumed more than 400 μg/day of dietary folic acid (*n* = 8,950) or used folic acid supplements daily (*n* = 15,976) during pregnancy. Table 5 shows the results of mothers who consumed more than 400 μg/day of dietary folic acid at mid-pregnancy. For communication, the lower intake groups of total dietary fiber had a higher risk of child developmental delay at the age of 3 years (Q1: aOR, 1.62; 95% CI, 1.05–2.50; *P* for trend = 0.004). For personal social skills, the second highest dietary fiber group had a lower risk of child developmental delay than did the highest dietary fiber group (Q4: aOR, 0.59; 95% CI, 0.36–0.96). Table 6 shows the results of mothers who used folic acid supplements daily during pregnancy. The group with the lowest total dietary fiber had a higher risk of child developmental delay at the age of 3 years in four domains than did the highest intake group: communication (Q1: aOR, 1.65; 95% CI, 1.23–2.19; *P* for trend < 0.001), fine motor skill (Q1: aOR, 1.54; 95% CI, 1.25–1.91; *P* for trend < 0.001), problem-solving (Q1: aOR, 1.54; 95% CI, 1.25–1.90; *P* for trend < 0.001), and personal-social skills (Q1: aOR, 1.44; 95% CI, 1.07–1.93; *P* for trend = 0.008).

**Table 5:** Association between intake of total dietary fiber and development delay at the age of 3 years in mothers with folic acid intake above the reference value (400 μg/day) during pregnancy.

**Subscales in ASQ-3**	**Communication**		**Gross motor**		**Fine motor**		**Problem solving**		**Personal-social**	
	**cOR (95% CI)**	**aOR (95% CI)**	**cOR (95% CI)**	**aOR (95% CI)**	**cOR (95% CI)**	**aOR (95% CI)**	**cOR (95% CI)**	**aOR (95% CI)**	**cOR (95% CI)**	**aOR (95% CI)**
**Total dietary fiber (g/1,000 kcal/day)**										
Q5 (≥9.93)	1.00	1.00	1.00	1.00	1.00	1.00	1.00	1.00	1.00	1.00
Q4 (8.33– < 9.93)	1.14 (0.76–1.73)	0.97 (0.60–1.56)	0.93 (0.67–1.30)	0.97 (0.66–1.42)	**0.76 (0.57–0.99)**	0.74 (0.54–1.02)	1.05 (0.80–1.39)	1.03 (0.75–1.43)	0.71 (0.47–1.08)	**0.59 (0.36–0.96)**
Q3 (7.11– < 8.33)	1.43 (0.97–2.13)	1.38 (0.89–2.15)	1.14 (0.83–1.56)	1.22 (0.84–1.76)	0.88 (0.68–1.15)	0.88 (0.65–1.21)	1.11 (0.85–1.46)	1.15 (0.84–1.58)	1.06 (0.72–1.55)	0.94 (0.61–1.45)
Q2 (5.78– < 7.11)	**1.68 (1.14–2.46)**	**1.66 (1.08–2.54)**	1.11 (0.81–1.52)	1.30 (0.91–1.87)	1.22 (0.95–1.56)	1.23 (0.92–1.64)	1.26 (0.96–1.64)	**1.43 (1.05–1.94)**	1.02 (0.69–1.50)	0.95 (0.61–1.45)
Q1 (< 5.78)	**1.73 (1.18–2.53)**	**1.62 (1.05–2.50)**	1.14 (0.83–1.56)	1.25 (0.86–1.81)	**1.34 (1.05–1.70)**	1.33 (1.00–1.78)	**1.34 (1.03–1.75)**	1.31 (0.95–1.79)	1.14 (0.78–1.66)	1.07 (0.69–1.64)
*P* for trend	**0.001**	**0.004**	0.264	0.096	**0.001**	**0.005**	**0.012**	**0.021**	0.257	0.442

cOR, crude odds ratio; aOR, adjusted odds ratio; CI, confidence interval; ASQ-3, Ages and Stages Questionnaires, Third Edition. Boldface indicates significance (*p* < 0.05).

Adjusted for maternal smoking and drinking during pregnancy, maternal age at birth, pre-pregnancy BMI, maternal and paternal education levels, annual household income, child sex, mode of delivery, birthweight, gestational age at birth, older siblings, attendance a childcare facility at 1 year of age, breastfeeding until 1 year of age, mother's K6 scores during pregnancy and when her child was 1 year of age, caretaker other than the child's mother at 1 year of age, and attachment bond when her child was 1 year of age.

**Table 6:** Association between intake of total dietary fiber and development delay at the age of 3 years in mothers who took daily folic acid supplements during pregnancy.

**Subscales in ASQ-3**	**Communication**		**Gross motor**		**Fine motor**		**Problem solving**		**Personal-social**	
	**cOR (95% CI)**	**aOR (95% CI)**	**cOR (95% CI)**	**aOR (95% CI)**	**cOR (95% CI)**	**aOR (95% CI)**	**cOR (95% CI)**	**aOR (95% CI)**	**cOR (95% CI)**	**aOR (95% CI)**
**Total dietary fiber (g/1,000 kcal/day)**										
Q5 (≥7.86)	1.00	1.00	1.00	1.00	1.00	1.00	1.00	1.00	1.00	1.00
Q4 (6.62– < 7.86)	1.25 (0.96–1.62)	1.32 (0.98–1.72)	1.02 (0.81–1.29)	1.00 (0.76–1.26)	1.09 (0.90–1.33)	1.09 (0.88–1.36)	1.16 (0.95–1.41)	1.16 (0.94–1.44)	1.17 (0.90–1.54)	1.15 (0.85–1.56)
Q3 (5.74– < 6.62)	**1.40 (1.08–1.81)**	**1.45 (1.09–1.94)**	**1.29 (1.03–1.61)**	**1.34 (1.05–1.72)**	**1.40 (1.16–1.68)**	**1.39 (1.12–1.72)**	**1.31 (1.06–1.55)**	**1.25 (1.01–1.55)**	1.24 (0.95–1.61)	1.23 (0.91–1.66)
Q2 (4.84– < 5.74)	**1.53 (1.19–1.97)**	**1.63 (1.23–2.17)**	1.12 (0.89–1.40)	1.15 (0.89–1.48)	**1.32 (1.09–1.59)**	**1.37 (1.10–1.70)**	**1.30 (1.09–1.60)**	**1.31 (1.05–1.62)**	1.30 (1.00–1.69)	**1.35 (1.00–1.82)**
Q1 (< 4.84)	**1.47 (1.14–1.89)**	**1.65 (1.23–2.19)**	1.09 (0.87–1.38)	1.12 (0.86–1.45)	**1.52 (1.26–1.83)**	**1.54 (1.25–1.91)**	**1.52 (1.26–1.83)**	**1.54 (1.25–1.90)**	**1.45 (1.12–1.88)**	**1.44 (1.07–1.93)**
*P* for trend	**0.001**	**< 0.001**	0.264	0.192	**< 0.001**	**< 0.001**	**< 0.001**	**< 0.001**	**0.004**	**0.008**

cOR, crude odds ratio; aOR, adjusted odds ratio; CI, confidence interval; ASQ-3, Ages and Stages Questionnaires, Third Edition. Boldface indicates significance (*p* < 0.05).

Adjusted for maternal smoking and drinking during pregnancy, maternal age at birth, pre-pregnancy BMI, maternal and paternal education levels, annual household income, child sex, mode of delivery, birthweight, gestational age at birth, older siblings, attendance a childcare facility at 1 year of age, breastfeeding until 1 year of age, mother's K6 scores during pregnancy and when her child was 1 year of age, caretaker other than the child's mother at 1 year of age, and attachment bond when her child was 1 year of age.

## 4. Discussion

Using a large birth cohort, we clarified for the first time the relationship between reduced maternal dietary fiber intake during pregnancy and increased neurodevelopmental risk in offspring, which had previously been reported in animal studies. Even after taking folic acid intake into account, there was a significant link between maternal dietary fiber intake during pregnancy and child developmental delay at the age of 3 years. Assessments at baseline and 1 year after study began showed that the study population was representative of the general population in Japan (14, 22), suggesting that the results of this study may be generalized.

The dietary reference intakes of the United States and Canada indicate that 28 g of total dietary fiber per day (14 g/1,000 kcal/day) is adequate for pregnant women (23). According to the dietary reference intake for Japanese (2020), the tentative dietary fiber intake goal for pregnant women is calculated to be 18 g or more per day (4). The Dietary Intake Standards for Japanese (2020 edition) recommend that pregnant women consume at least 18 g of dietary fiber per day. In this study, the mean (± SD) of maternal dietary fiber intake during pregnancy was 10.77 (± 6.30) g per day, with only 6,391 mothers (8.4%) consuming more than 18 g per day. Therefore, we used the group with the highest dietary fiber intake as a reference. The lower the dietary fiber intake, the higher the odds ratio for child developmental delay at the age of 3 years.

Animal studies have shown that offspring born to mothers fed a low-fiber diet during pregnancy are at a higher risk for metabolic syndrome, characterized by obesity and impaired glucose tolerance (9). In contrast, maternal high-fiber diets have been demonstrated to reduce cognitive and social-behavioral dysfunction in offspring caused by maternal obesity (10). This study corroborates findings from animal studies, and extends them by demonstrating the link between maternal dietary fiber intake during pregnancy and the risk of neurodevelopmental delay in offspring.

The primary mechanism by which dietary fiber acts is by regulating the gut microbiome. Murine studies have demonstrated a link between the dysbiosis of the maternal gut microbiome during pregnancy and abnormal neurodevelopmental and behavior in offspring (24, 25). In addition, an Australian birth cohort study recently found a link between maternal gut microbiota composition during pregnancy and behavioral abnormalities at age two (26). Specifically, recent studies have demonstrated that short-chain fatty acids (SCFAs) produced by bacterial fermentation of dietary fiber are important for gut microbiota-brain crosstalk (27, 28). Short-chain fatty acids such as acetate, propionate, and butyrate are known to modulate sympathetic nervous system activation (9) and affect brain function and behavior (12, 29). Our results suggest that maternal inadequate dietary fiber intake during pregnancy affected child neurodevelopmental delay through decreased production of SCFAs by gut bacterial fermentation of dietary fiber. Soluble and insoluble dietary fiber had similar effects on the four ASQ-3 subscales (communication, fine motor, problem-solving, and personal-social). However, only soluble dietary fiber was found to significantly increase the risk of child developmental delay in gross motor. Soluble dietary fiber is known to affect the regulation of gut microbiota and the production of SCFAs compared to that by insoluble dietary fiber (30). Thus, maternal soluble fiber intake during pregnancy may have significantly impacted child development.

The gut microbiota composition of pregnant women with overweight is known to differ from that of pregnant women with normal weight (31, 32). The gut microbiota of women with maternal pre-pregnancy overweight has also been shown to influence the gut microbiota composition of their offspring (33, 34). In recent years, it has been clarified that the gut microbiota may influence the association between maternal overweight and neurodevelopmental abnormalities in children (35–37). Therefore, to exclude the effects of maternal overweight, we investigated the association between total dietary fiber intake and developmental delay at the age of 3 years, stratified by pre-pregnancy BMI. Our results showed that even in the normal weight group, lower dietary fiber intake during pregnancy was associated with an increased risk of child developmental delay in four ASQ-3 subscales (communication, fine motor, problem-solving, and personal-social). These results strengthen the evidence that inadequate intake of dietary fiber during pregnancy affects neurodevelopmental delay in offspring. Therefore, future studies should elucidate the molecular mechanisms associated with maternal dietary fiber intake during pregnancy and neurodevelopment of the offspring by analyzing the gut microbiota and its metabolites.

Grains, beans, potatoes, vegetables, fruits, mushrooms, and seaweed are known as food groups rich in dietary fiber (38). Therefore, our results may reflect the effects of other nutrients that are highly correlated with dietary fiber intake. Folic acid deficiency during early pregnancy is known to be associated with an increased risk of neural tube defects and neurodevelopmental disorders, such as autism (39, 40). A JECS study found a link between dietary folic acid intake by pregnant women and child's verbal cognitive development at 2 years of age (41). According to the dietary reference intakes for Japanese (2020), the estimated average folic acid requirement for pregnant women is 400 μg per day (4). However, only 8,950 (11.7%) pregnant women in this study satisfied the requirements for dietary folic acid intake. In fact, when examining the association between intake of dietary fiber and various nutrients, folic acid showed the highest correlation (*r* = 0.865). To eliminate the effect of folic acid, we analyzed the link between maternal dietary fiber intake during pregnancy and child developmental delay at the age of 3 years in a group of pregnant women with folic acid intake above the reference value. As a result, similar effects were observed in communication, fine motor, and problem-solving. In fine motor and personal-social, the group with the second highest dietary fiber intake (Q2) had a reduced odds ratio compared with the group with the highest dietary fiber intake (Q1), which may reflect the effect of excessive dietary fiber intake. Therefore, supplementation is recommended since it is difficult to obtain sufficient folic acid from food (4). Our results also showed a link between maternal dietary fiber intake during pregnancy and child developmental delay, in an analysis of a population of mothers taking daily folic acid supplements. Many of the folic acid supplements for pregnant women sold in Japan also contain various vitamins, minerals, iron, and calcium. Therefore, it is conceivable that mothers who take folic acid supplements appropriately also take sufficient nutrients necessary for pregnant women. The results of these sensitivity analyses strengthen the evidence that lower maternal dietary fiber intake during pregnancy is linked to a higher risk of child neurodevelopmental delay. Therefore, it may be necessary to recommend dietary fiber intake through supplementation in pregnant women.

The strength of this study is its large sample size and adjustment for many potential confounders. Our results provided reinforcing evidence for the DOHaD concept that undernutrition during pregnancy is associated with an increased risk of neurodevelopmental delay in children. Nevertheless, this study had some limitations. First, human studies cannot assess the effects of dietary fiber alone. Although this study considered the impact of folic acid intake during pregnancy, the possibility of other nutrients having an impact cannot be completely ruled out. In addition, dietary fiber intake from supplements could not be investigated. Therefore, further intervention studies are needed to clarify the direct impact. Second, because this study used a self-administered questionnaire, it is possible that personal understanding of the questions influenced responses. Third, the FFQ we used has not been validated in pregnant women and may be misclassified. Finally, regarding the nutritional environment after childbirth, breastfeeding was adjusted; however, baby food and early childhood diet were not considered.

This study indicates that maternal dietary fiber deficiency during pregnancy may contribute to a higher risk of neurodevelopmental delay in offspring. Furthermore, most pregnant women in Japan consume far less dietary fiber than what is the recommended intake; thereby, this maternal nutritional imbalance during pregnancy may adversely affect the neurodevelopment of their offspring. Therefore, nutritional guidance for pregnant mothers is crucial to reduce the risk of future health problems for their children.

## Data availability statement

The datasets presented in this article are not readily available because data are unsuitable for public deposition due to ethical restrictions and legal framework of Japan. It is prohibited by the Act on the Protection of Personal Information (Act No. 57 of 30 May 2003, amendment on 9 September 2015) to publicly deposit the data containing personal information. Ethical Guidelines for Medical and Health Research Involving Human Subjects enforced by the Japan Ministry of Education, Culture, Sports, Science and Technology and the Ministry of Health, Labour and Welfare also restricts the open sharing of the epidemiologic data. All inquiries about access to data should be sent to: jecs-en@nies.go.jp. The person responsible for handling enquiries sent to this e-mail address is Dr. Shoji F. Nakayama, JECS Programme Office, National Institute for Environmental Studies. Requests to access the datasets should be directed to Dr. Shoji F. Nakayama, jecs-en@nies.go.jp/b.

## Ethics statement

The studies involving human participants were reviewed and approved by the Ministry of the Environment's Institutional Review Board on Epidemiological Studies and the Ethics Committees. Written informed consent to participate in this study was provided by the participants' legal guardian/next of kin.

## Author contributions

KMi conceived and designed the study, drafted the initial manuscript, and revised the manuscript. SH, RS, MK, SO, ZY, and the JECS Group collected data and critically reviewed and revised the manuscript. HYu, YA, TO, RK, HYo, and KMo critically reviewed the manuscript. All authors approved the final manuscript as submitted and agree to be accountable for all aspects of the work.

## Group members of The Japan Environment and Children's Study Group

Members of the JECS Group as of 2022: Michihiro Kamijima (principal investigator, Nagoya City University, Nagoya, Japan); Shin Yamazaki (National Institute for Environmental Studies, Tsukuba, Japan); Yukihiro Ohya (National Center for Child Health and Development, Tokyo, Japan); Reiko Kishi (Hokkaido University, Sapporo, Japan); Nobuo Yaegashi (Tohoku University, Sendai, Japan); Koichi Hashimoto (Fukushima Medical University, Fukushima, Japan); Chisato Mori (Chiba University, Chiba, Japan); Shuichi Ito (Yokohama City University, Yokohama, Japan); Zentaro Yamagata (Universit y of Yamanashi, Chuo, Japan); Hidekuni Inadera (University of Toyama, Toyama, Japan); Takeo Nakayama (Kyoto University, Kyoto, Japan); Tomotaka Sobue (Osaka University, Suita, Japan); Masayuki Shima (Hyogo Medical University, Nishinomiya, Japan); Hiroshige Nakamura (Tottori University, Yonago, Japan); Narufumi Suganuma (Kochi University, Nankoku, Japan); Koichi Kusuhara (University of Occupational and Environmental Health, Kitakyushu, Japan); and Takahiko Katoh (Kumamoto University, Kumamoto, Japan).
